# Maxillary reconstruction using tunneling flap technique with 3D custom-made titanium mesh plate and particulate cancellous bone and marrow graft: a case report

**DOI:** 10.1186/s40902-019-0228-y

**Published:** 2019-10-17

**Authors:** Masayuki Takano, Keisuke Sugahara, Masahide Koyachi, Kento Odaka, Satoru Matsunaga, Shinya Homma, Shinichi Abe, Akira Katakura, Takahiko Shibahara

**Affiliations:** 1 Department of Oral and Maxillofacial Surgery, Tokyo Dental College, 2-9-18 Kandamisaki-cho, Chiyoda-ku, Tokyo, 101-0061 Japan; 2 Department of Oral Pathobiological Science and Surgery, Tokyo Dental College, 2-9-18 Kandamisaki-cho, Chiyoda-ku, Tokyo, 101-0061 Japan; 3 Oral Health Science Center, Tokyo Dental College, 2-9-18 Kandamisaki-cho, Chiyoda-ku, Tokyo, 101-0061 Japan; 4 Department of Oral and Maxillofacial Radiology, Tokyo Dental College, 2-9-18 Kandamisaki-cho, Chiyoda-ku, Tokyo, 101-0061 Japan; 5 Department of Anatomy, Tokyo Dental College, 2-9-18 Kandamisaki-cho, Chiyoda-ku, Tokyo, 101-0061 Japan; 6 Department of Oral and Maxillofacial Implantology, Tokyo Dental College, 2-9-18 Kandamisaki-cho, Chiyoda-ku, Tokyo, 101-0061 Japan

**Keywords:** Mouth neoplasms, Reconstructive surgical procedure, Three-dimensional printing, Surgical flap

## Abstract

**Background:**

Reconstructive surgery is often required for tumors of the oral and maxillofacial region, irrespective of whether they are benign or malignant, the area involved, and the tumor size. Recently, three-dimensional (3D) models are increasingly used in reconstructive surgery. However, these models have rarely been adapted for the fabrication of custom-made reconstruction materials. In this report, we present a case of maxillary reconstruction using a laboratory-engineered, custom-made mesh plate from a 3D model.

**Case presentation:**

The patient was a 56-year-old female, who had undergone maxillary resection in 2011 for intraoral squamous cell carcinoma that presented as a swelling of the anterior maxillary gingiva. Five years later, there was no recurrence of the malignant tumor and a maxillary reconstruction was planned. Computed tomography (CT) revealed a large bony defect in the dental-alveolar area of the anterior maxilla. Using the CT data, a 3D model of the maxilla was prepared, and the site of reconstruction determined. A custom-made mesh plate was fabricated using the 3D model (Okada Medical Supply, Tokyo, Japan). We performed the reconstruction using the custom-made titanium mesh plate and the particulate cancellous bone and marrow graft from her iliac bone. We employed the tunneling flap technique without alveolar crest incision, to prevent surgical wound dehiscence, mesh exposure, and alveolar bone loss. Ten months later, three dental implants were inserted in the graft. Before the final crown setting, we performed a gingivoplasty with palate mucosal graft. The patient has expressed total satisfaction with both the functional and esthetic outcomes of the procedure.

**Conclusion:**

We have successfully performed a maxillary and dental reconstruction using a custom-made, pre-bent titanium mesh plate.

## Background

Oral and maxillofacial reconstructive surgery provides optimal structural and functional rehabilitation for people with congenital anomalies, tumors, and trauma involving the maxillary and mandibular area. Irrespective of whether a tumor is benign or malignant, reconstructive surgery is often required based on the area of involvement and tumor size. Recently, various surgical specialties, including oral and maxillofacial surgery, employ three-dimensional (3D) models for reconstruction surgery [1–3]. However, these models have been rarely adapted for the fabrication of custom-made reconstruction materials [4, 5]. In this report, we present a case of mandibular reconstruction using a custom-made titanium mesh plate. The mesh plate was made in a laboratory from the computed tomography (CT)-generated 3D model of a patient’s maxillary area. We also used alternative surgical techniques to prevent postoperative wound dehiscence.

## Case report

**Fig. 1:**
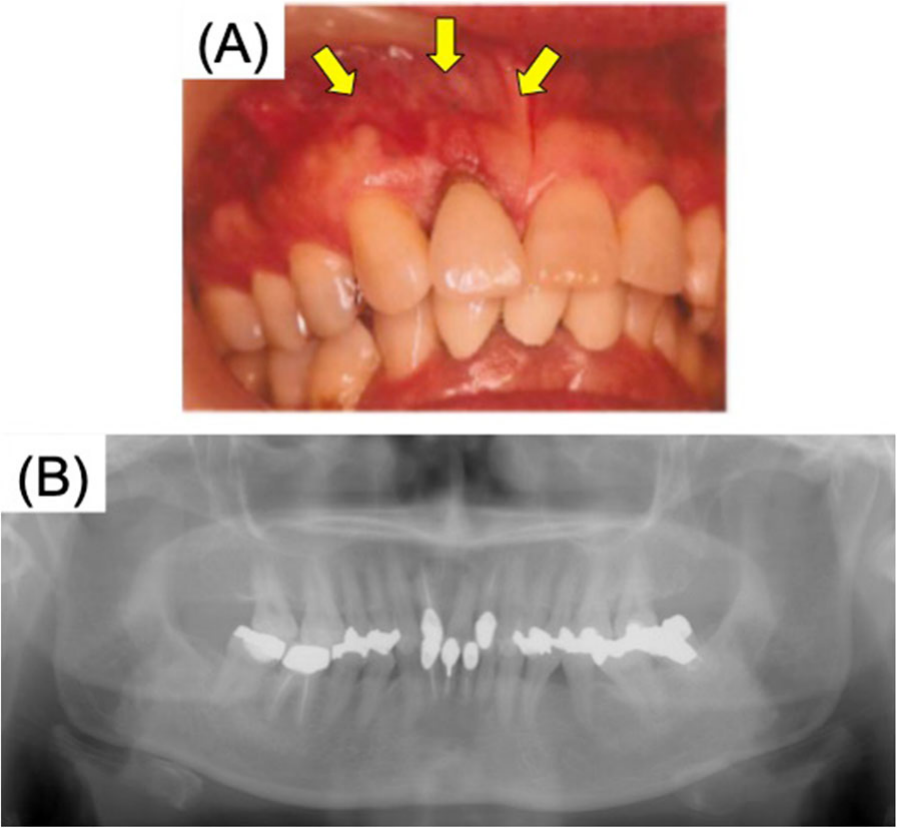
First examination showing swelling (arrow) diagnosed as gingival SCC of the maxilla. **a** Intraoral findings. **b** Preoperative radiograph. SCC, squamous cell carcinoma

**Fig. 2:**
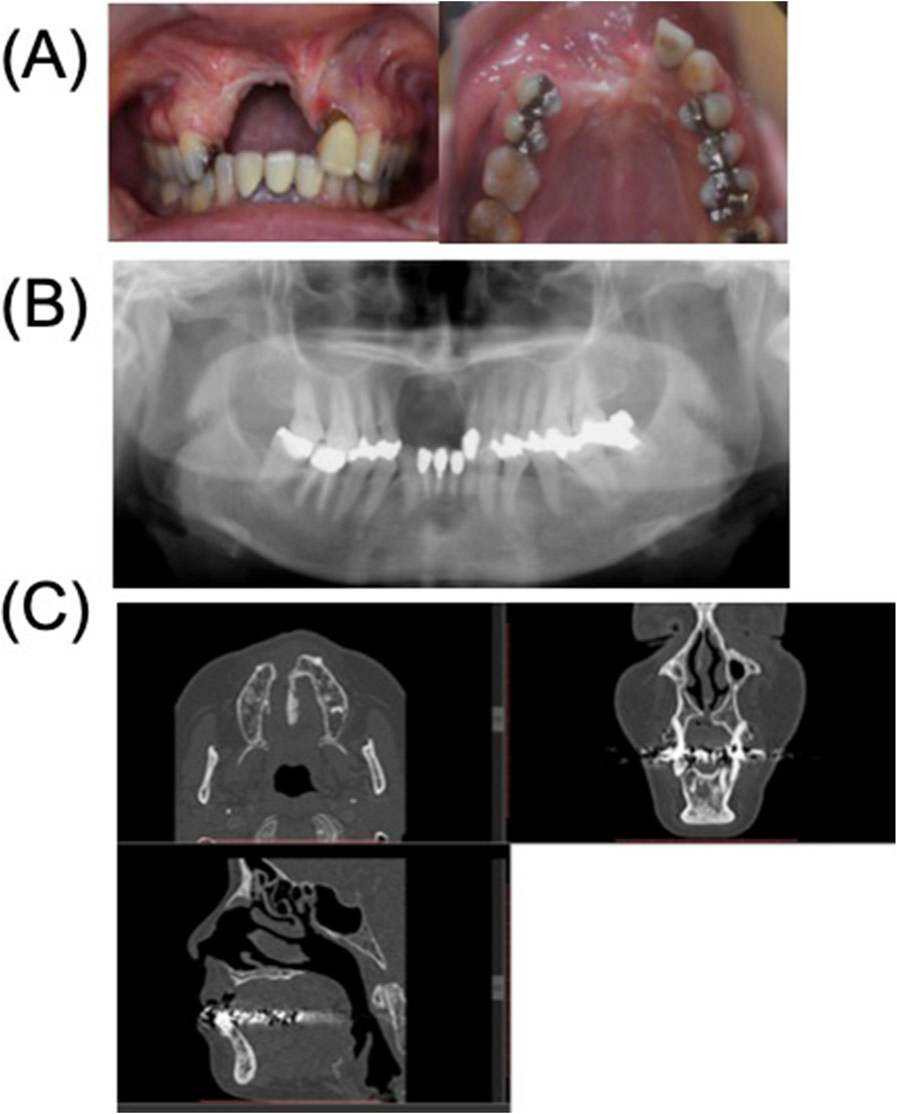
Findings after partial resection of the maxilla. **a** Intraoral findings showing abundant scar tissue formation. **b** Postoperative radiograph. **c** Postoperative cone-beam CT showing segmental defect in the anterior maxilla

**Fig. 3:**
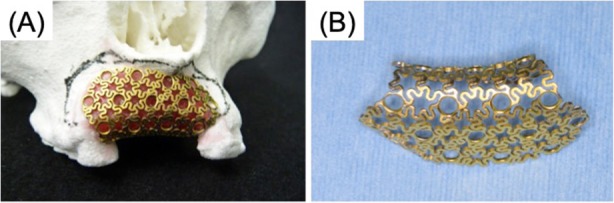
3D custom-made titanium mesh plate. **a** 3D custom plate was made on 3D model. **b** Occlusal view

The patient was a 56-year-old female, who presented with the chief complaint of swelling in the anterior upper gingiva in 2011 (Fig. 1). On examination and biopsy, she was diagnosed with intraoral squamous cell carcinoma (T1N0M0) for which a maxillary resection was performed. Immediate reconstruction was not adopted because of the large defect in the alveolar mucosa and difficulty with postoperative follow-up. Five years later, there was no recurrence of the malignant tumor, and hence, a maxillary reconstruction was planned. On examination, the patients’ facial appearance was symmetrical, but without the prosthesis, her upper lip recessed. CT revealed a large bony defect involving the dental-alveolar area in the anterior maxilla (Fig. 2). Using the data from the CT, we prepared a 3D model of the maxilla using a 3D printer (Stratasys Co. Connex 260) and the site of reconstruction was determined. Then, a custom-made mesh titanium plate was fabricated in the remote laboratory, based on the 3D model (ULTRA FLEX MESH CUSTOM, Okada Medical Supply, Tokyo, Japan) (Fig. 3). This plate thickness is 0.6 mm. And the titanium mesh plate was given a basic hexagonal polygonal shape designed to have high 3D flexibility.

**Fig. 4:**
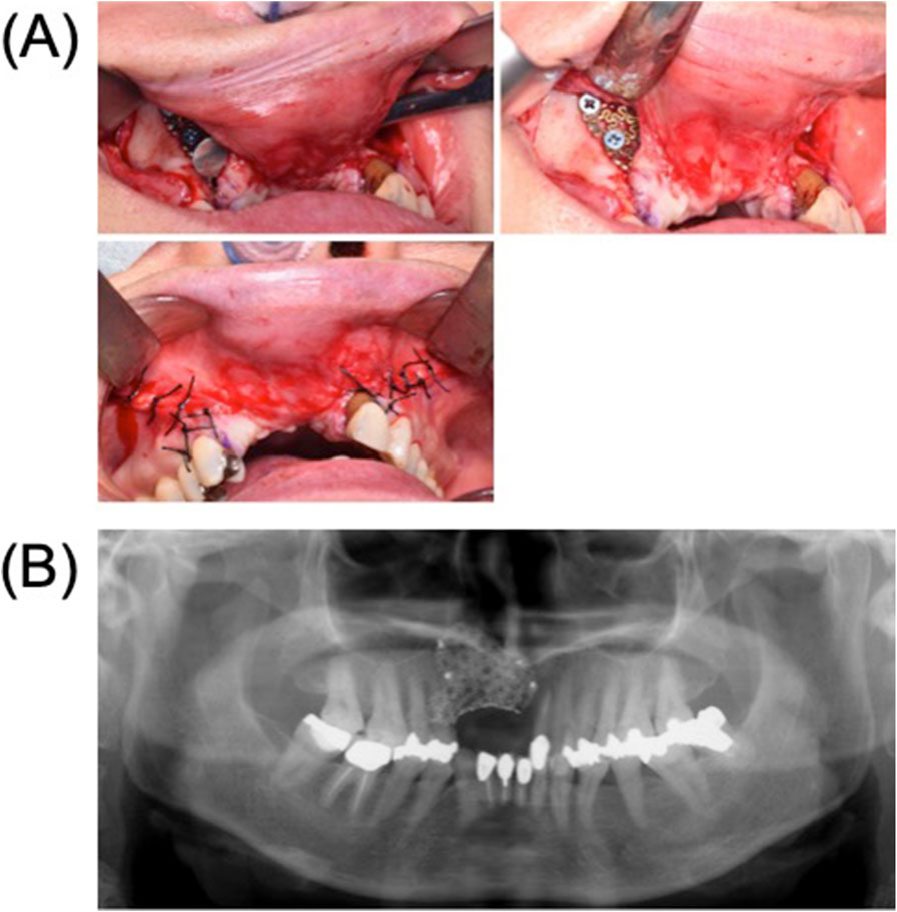
Reconstruction with a custom-made titanium mesh by tunneling flap technique. **a** Intraoperative oral photograph. **b** Postoperative radiograph

We used the custom-made titanium mesh plate and particulate cancellous bone and marrow graft (PCBM) from her iliac bone to reconstruct the maxillary defect. To prevent surgical wound dehiscence and subsequent exposure of the mesh plate and alveolar bone loss, the custom-made mesh plate was inserted using the tunneling flap technique as opposed to the alveolar crest incision. The mesh plate was fixed to the maxilla using several bone screws, following which PCBM was used to fill the inside of the mesh. Finally, we closed the surgical incision by suturing (Fig. 4). The total operative time was 118 min, and total blood loss was 50 ml. No adverse events occurred during the surgery.

**Fig. 5:**
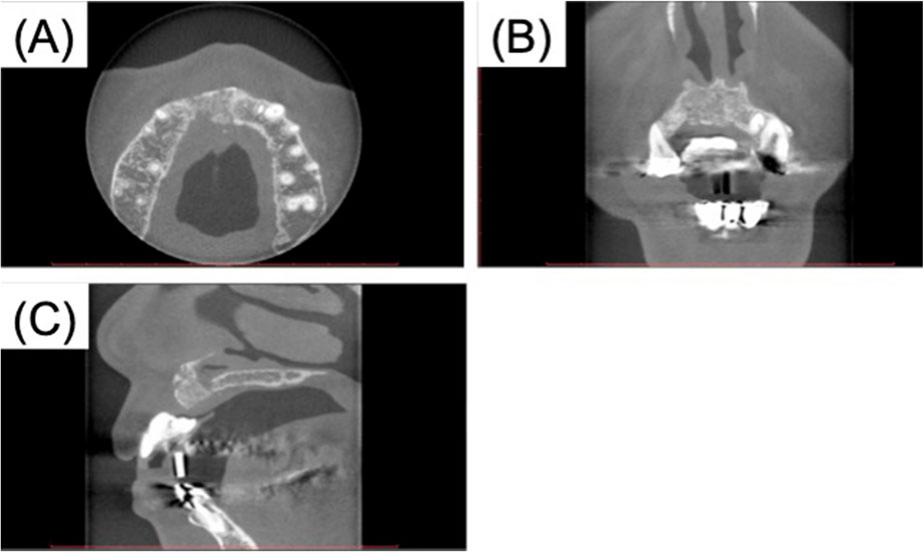
Cone-beam CT findings after removal of the mesh. **a** Axial. **b** Coronal. **c** Sagittal

**Fig. 6:**
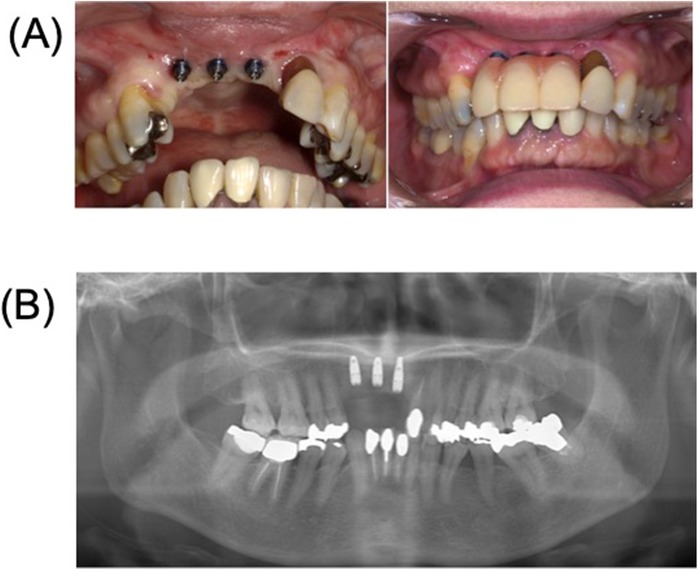
Placement of three dental implants and provisional prosthesis. **a** Intraoral findings. **b** Postoperative radiograph

**Fig. 7:**
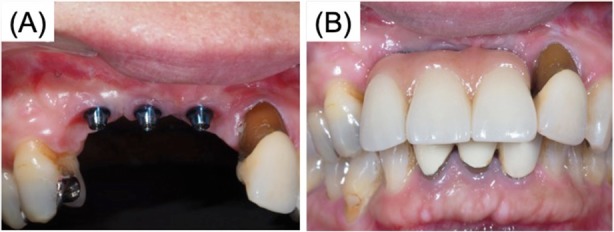
After gingivoplasty with palate graft for prevention of peri-implantitis. **a** Intraoral findings after gingivoplasty. **b** Intraoral findings, final prosthesis

Seven months after the reconstruction, we removed the mesh plate and performed a vestibuloplasty with an atelocollagen sheet (TERUDERMIS™, OLYMPUS TERUMO BIOMATERIALS, Tokyo, Japan). (Fig. 5). Ten months after reconstruction, three dental implant fixtures (diameter 3.3 mm, SLActive, Straumann Japan) were inserted in the graft (Fig. 6). Before the final crown setting, we performed an additional gingivoplasty using palate mucosal graft (Fig. 7). The patient has been completely satisfied with the treatment, both functionally and esthetically. This case report has been approved by the ethics committee of Tokyo Dental College (no. 646) (Tokyo, Japan).

## Discussion

Many institutions have recently started using 3D printers to create 3D models for various diseases in the field of oral and maxillofacial surgery. The first medical fabrication laboratory in Japan was established at Tokyo Dental College, “the Fab Lab TDC,” in December 2013 [1]. The laboratory utilizes various techniques to construct 3D models of the jaw, based on both CT and magnetic resonance imaging (MRI) images [1–3]. In the past, we had created 3D-printed models of jaw deformities and maxillary and mandibular tumors, and used them primarily during a consultation as preoperative simulations in patients. Globally, customized metal plates for jaw deformity and its reconstruction are made using computer-aided design/computer-aided manufacturing (CAD/CAM), based on preoperative computer simulations [6–8].

In our case, we successfully retained the shape and the function of the maxilla following a large resection by using a 3D custom-made titanium mesh plate and PCBM. The fabrication of custom-made products required CT data of the patient, and the relevant images were sent to FabLab TDC. The FabLab TDC used a 3D printer and Mimics software (Materialize NV, Leuven, Belgium) to construct a 3D model. Artifacts were removed manually using a computer. During conventional reconstruction, the surgeon bends the 3D mesh plate during surgery, whereas the current technique has the advantage of using a pre-bent, 3D mesh plate that is much easier to fix intraoperatively. The overall process of fabrication is simple and fast and has widespread applications in oral and maxillofacial surgery. This advancement in medical technology may lead to significant improvement in surgical care and outcome.

Titanium mesh was first used in 1985 for the reconstruction of atrophic alveoli [9]. Boyne et al. reported contouring the titanium mesh over the edentulous dental models to restore it to a more ideal ridge form [9]. The use of titanium mesh with particulate bone graft has several advantages [10]. The main disadvantages of a titanium mesh plate were the cost and the high rate of plate exposure [11]. In particular, when a large augmentation is planned, a vestibular incision may increase the risk of wound dehiscence. In our case, following the tumor resection, the mucosal wound healing was weak. As a result, we decided to employ a minimally invasive subperiosteal tunneling flap technique [12, 13]. The severe adhesion and fibrosis were left behind the maxillary resection. We made it possible to use a titanium mesh plate by inserting a long incision on the labial side and a cervical incision on the palate side. And paying attention to the blood supply of the mucous membrane, peritoneal-releasing incisions were made at the same time. The use of a pre-bent 3D titanium mesh and the tunneling flap makes the surgery much easier and simpler, and subsequently improves the accuracy and safety. It has been previously reported that the use of a custom-made mesh plate for maxillary reconstruction shortens the surgery time [4, 5].

## Conclusion

We have successfully performed a maxillary and dental reconstruction, using a 3D custom-made, pre-bent titanium mesh plate, fabricated from a 3D model which was generated from CT data. The tunneling flap technique for titanium mesh insertion is ideal for cases predisposed to post-surgical wound dehiscence, graft exposure and infection, and alveolar bone loss. The use of a pre-bent titanium mesh with tunneling flap incision reduces surgery time and improves safety.

## Data Availability

None.
